# Electric field-driven microfluidics for rapid CRISPR-based diagnostics and its application to detection of SARS-CoV-2

**DOI:** 10.1073/pnas.2010254117

**Published:** 2020-11-04

**Authors:** Ashwin Ramachandran, Diego A. Huyke, Eesha Sharma, Malaya K. Sahoo, ChunHong Huang, Niaz Banaei, Benjamin A. Pinsky, Juan G. Santiago

**Affiliations:** ^a^Department of Aeronautics & Astronautics, Stanford University, Stanford, CA 94305;; ^b^Department of Mechanical Engineering, Stanford University, Stanford, CA 94305;; ^c^Department of Biochemistry, Stanford University, Stanford, CA 94305;; ^d^Department of Clinical Pathology, Stanford University, Stanford, CA 94305;; ^e^Department of Medicine, Division of Infectious Diseases and Geographic Medicine, Stanford University, Stanford, CA 94305

**Keywords:** COVID-19, microfluidics, CRISPR diagnostics, isotachophoresis, rapid testing

## Abstract

The rapid spread of COVID-19 across the world has revealed major gaps in our ability to respond to new virulent pathogens. Rapid, accurate, and easily configurable molecular diagnostic tests are imperative to prevent global spread of new diseases. CRISPR-based diagnostic approaches are proving to be useful as field-deployable solutions. In one basic form of this assay, the CRISPR–Cas12 enzyme complexes with a synthetic guide RNA (gRNA). This complex becomes activated only when it specifically binds to target DNA and cleaves it. The activated complex thereafter nonspecifically cleaves single-stranded DNA reporter probes labeled with a fluorophore−quencher pair. We discovered that electric field gradients can be used to control and accelerate this CRISPR assay by cofocusing Cas12–gRNA, reporters, and target within a microfluidic chip. We achieve an appropriate electric field gradient using a selective ionic focusing technique known as isotachophoresis (ITP) implemented on a microfluidic chip. Unlike previous CRISPR diagnostic assays, we also use ITP for automated purification of target RNA from raw nasopharyngeal swab samples. We here combine this ITP purification with loop-mediated isothermal amplification and the ITP-enhanced CRISPR assay to achieve detection of severe acute respiratory syndrome coronavirus 2 (SARS-CoV-2) RNA (from raw sample to result) in about 35 min for both contrived and clinical nasopharyngeal swab samples. This electric field control enables an alternate modality for a suite of microfluidic CRISPR-based diagnostic assays.

## Significance

Rapid, early-stage screening is crucial during pandemics for early identification of infected patients and control of disease spread. CRISPR biology offers new methods for rapid and accurate pathogen detection. Despite their versatility and specificity, existing CRISPR diagnostic methods suffer from the requirements of up-front nucleic acid extraction, large reagent volumes, and several manual steps—factors which prolong the process and impede use in low-resource settings. We here combine microfluidics, on-chip electric field control, and CRISPR to directly address limitations of current CRISPR diagnostic methods. We apply our method to the rapid detection of SARS-CoV-2 RNA in clinical samples. Our method takes about 35 min from raw sample to result, a significant improvement over existing nucleic acid-based diagnostic methods for COVID-19.

Infectious diseases such as COVID-19 are a persistent global threat. Early-stage screening and rapid identification of infected patients are important during pandemics to treat the infected and to control disease spread. The frontline diagnostic tool for COVID-19 has been RT-PCR, and protocols for this have been developed and published by the World Health Organization (WHO) (1) and the US Centers for Disease Control and Prevention (CDC) (2). While these tests are specific and sensitive, they are laborious and time consuming, and are designed for large, centralized diagnostic laboratories.

CRISPR-based diagnostic methods, including diagnostic assays for the COVID-19 pandemic (3, 4), have sparked great interest due to their versatility, sensitivity, and specificity. CRISPR applications for infectious disease diagnostics take advantage of a nuclease-like “collateral cleavage” induced by Cas12 and Cas13 enzymes (5, 6). In such methods, RNA-guided CRISPR-associated proteins such as Cas12 and Cas13 can be programmed to detect specific DNA and RNA sequences, respectively, from pathogens with single base pair specificity. For CRISPR–Cas12-based diagnostics, the CRISPR complex between the Cas12 enzyme and the synthetic guide RNA (gRNA) first recognizes and specifically cleaves (known as *cis*-cleavage) target pathogen single-stranded DNA (ssDNA) and/or double-stranded DNA (dsDNA) in the sample. The gRNA is designed to have between 18 and 24 nucleotides complementary to the target DNA sequence. This molecular recognition modifies the Cas12-gRNA complex into its “activated” form, which thereafter indiscriminately cleaves ssDNA molecules including synthetic ssDNA reporter molecules with fluorophore−quencher pairs. The nuclease-like characteristic of Cas12a on ssDNA, known as *trans-*cleavage, is activated only in the presence of target ssDNA or dsDNA activator (5). Thus, recognition of target DNA by Cas12a results in an increase in fluorescence signal due to the *trans*-cleavage activity of Cas12a on reporter molecules, and this recognition feature makes CRISPR useful for diagnostic applications.

Despite advantages, several factors have impeded automated CRISPR-based detection methods. For example, the CRISPR–Cas12a-based method for severe acute respiratory syndrome coronavirus 2 (SARS-CoV-2) developed by Broughton et al. (3) in March 2020 required upfront nucleic acid extraction and sample purification with a traditional adsorption/desorption column for purification, a process which typically takes up to 1 h. Moreover, Broughton et al. (3) carried out CRISPR enzymatic reactions in Eppendorf tubes and explored both colorimetric (using a lateral flow strip) and fluorescence readouts for target detection. Such protocols are not easily amenable to automation, consume significant reagent volume [typical CRISPR transcleavage assays are carried out in 50 to 100 μL volumes (3, 4)], and require 1 h or longer to complete, starting from raw sample. The consumption of reagents is important. For example, Joung et al. (4) reported supply chain constraints in procuring RPA (recombinase polymerase amplification) reagents for a CRISPR–Cas13-based test which they initially developed for SARS-CoV-2 in February 2020. This limitation compelled them to redesign their assay to one based on CRISPR–Cas12b and loop-mediated isothermal amplification (LAMP) in May 2020.

Microfluidics offers important alternate strategies to accelerate biochemical reactions (7), multiplex (8), and automate CRISPR diagnostics. We here develop an electric field-enhanced microfluidic method that is broadly applicable to the field of CRISPR diagnostics. To this end, we use an electrokinetic microfluidic technique called isotachophoresis (ITP). ITP uses a two-buffer system which consists of a high-mobility leading electrolyte (LE) and a low-mobility trailing electrolyte (TE) buffer. On application of an electric field, sample ions with effective mobilities bracketed by the LE and TE ions selectively focus within an order 10 μm zone at the LE-to-TE interface. This focusing can preconcentrate, purify, mix, and accelerate reactions among sample and reagents species. ITP has been used to rapidly extract nucleic acids from a range of biological samples such as urine (9), blood (10), and cell lysates (11), and to accelerate DNA and RNA hybridization reactions (12).

For ITP applications involving purification and extraction of nucleic acids, a proper choice of LE and TE ensures that target species (here, DNA and RNA) focus and preconcentrate in ITP, while leaving behind impurities and inhibitors to downstream analyses (here, inhibitors can include proteins and small cations) (10, 11). See Rogacs et al. (11) for a detailed review on purification of nucleic acids using ITP. When ITP is applied to control homogeneous biochemical reactions, a good choice of LE and TE enables all reacting species to cofocus and preconcentrate in ITP. The simultaneous preconcentration of all reactants in ITP accelerates product formation. As an example, Bercovici et al. (12) used ITP to demonstrate 14,000-fold acceleration of DNA hybridization assays. See Eid and Santiago (7) for a comprehensive review on ITP-enhanced biochemical reactions, including both homogeneous and heterogenous reactions.

In this work, we combine microfluidics and on-chip electric field control to achieve two critical steps. First, we use ITP to automatically extract nucleic acids from raw biological samples, here, nasopharyngeal (NP) swab samples from COVID-19 patients and healthy controls. Second, we use electric field gradients in ITP to control and effect rapid CRISPR–Cas12 enzymatic activity upon target nucleic acid recognition. The latter is achieved using a tailored on-chip ITP process to cofocus Cas12–gRNA, reporter ssDNA, and target nucleic acids (*SI Appendix*, Figs. S1 and S2). This creates simultaneous mixing, preconcentration, and acceleration of enzymatic reactions. Our microfluidic method consumes minimal volume of reagents (order 100-fold lower than conventional methods) on-chip for CRISPR reactions and is amenable to automation. We apply our method to detection of SARS-CoV-2 RNA in an assay which takes around 30 min to 40 min from raw sample to result. We demonstrate this on clinical samples, including SARS-CoV-2 positive and negative clinical specimens. The method is both an alternate modality for CRISPR diagnostics and, to our knowledge, the fastest CRISPR-based detection of SARS-CoV-2 from raw samples with clinically relevant specificity and sensitivity.

## Results

### Microfluidic ITP-CRISPR−Based Protocol for Rapid SARS-CoV-2 Detection from Raw NP Swab Samples.

**Fig. 1.**
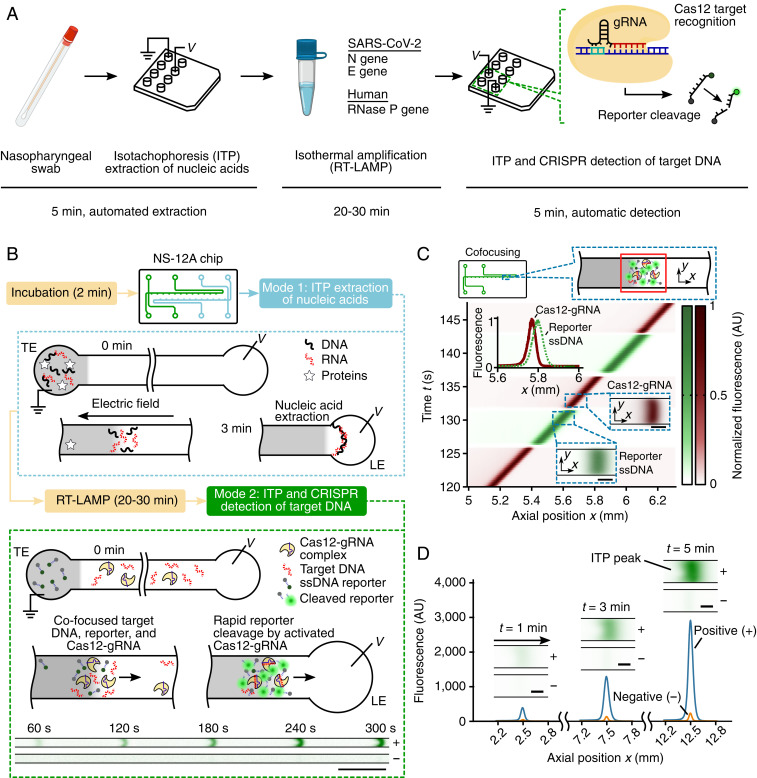
An electric field-mediated microfluidic assay for SARS-CoV-2 RNA detection using ITP and CRISPR–Cas12. (*A*) Schematic of SARS-CoV-2 detection workflow from sample to result. Microfluidic ITP is used to extract nucleic acids from raw NP sample, followed by off-chip RT-LAMP preamplification and on-chip ITP-CRISPR−based fluorescent detection of N, E, and RNase P genes. CRISPR–Cas12 activation by the presence of target cDNA of SARS-CoV-2 results in nonspecific ssDNA cleavage and unquenching of a reporter ssDNA labeled with a fluorophore and quencher (5). (*B*) Assay working principle. A single microfluidic chip with two channels is used for ITP extraction of nucleic acids (mode 1) and ITP–CRISPR detection (mode 2). In mode 1 (within dotted blue rectangle), on application of an electric field, nucleic acids with electrophoretic mobility bracketed by the leading (LE) and trailing (TE) electrolyte ions selectively focus within the electromigrating LE–TE interface, leaving behind impurities (10, 11). Following off-chip RT-LAMP of ITP-extracted nucleic acids, in mode 2 (within the green dashed rectangle), ITP is used to effect target DNA detection using a CRISPR-Cas12 enzyme assay. A positive sample shows a strong fluorescent signal compared to the negative control. (Scale bar, 0.5 mm.) (*C*) Electric field control of Cas12–gRNA and nucleic acids. Experimental visualization of the moving ITP interface in mode 2 using a fluorescently tagged gRNA (red) and ssDNA reporter (green). Spatiotemporal intensity plots of the green and red fluorescence emission show that Cas12–gRNA and nucleic acids electromigrate and cofocus in a ∼100-pL ITP interface volume. *Top Inset* shows ssDNA fluorescence intensity profile (green) at 135 s and comparison with the Cas12–gRNA profile (red). *Bottom Insets* show instantaneous fluorescence images of the ITP peak which includes labeled Cas12–gRNA (red) and reporter ssDNA (green). (Scale bar, *Bottom Insets*, 50 μm.) (*D*) Example quantitative measurements of on-chip fluorescence detection from cleaving of quencher/fluorophore ssDNA reporters by ITP-focused and activated Cas12–gRNA complex. Raw on-chip fluorescence signal versus axial location for ITP-CRISPR detection of E gene of SARS-CoV-2 positive and negative controls in mode 2. *Insets* show instantaneous epifluorescence microscopy images of the moving ITP interface. (Scale bar, 50 μm.)

We developed and optimized a microfluidic protocol to rapidly detect SARS-CoV-2 viral RNA in around 30 min starting from raw NP swab samples in viral transport medium (VTM) (Fig. 1*A*). After a 2-min preincubation step (at 62 °C) of raw NP sample with lysis buffer, we leverage on-chip ITP to rapidly extract total nucleic acids (both host DNA and any viral RNA) from the lysed sample in 3 min (Fig. 1*B*; mode 1). Next, RT-LAMP isothermal amplification (20 min to 30 min) at 62 °C is performed off-chip on the ITP extract using a water bath, targeting the viral N and E genes and human RNase P genes in separate reactions. In the last step of our protocol, we use ITP to perform rapid (<5 min), on-chip CRISPR–Cas12-based enzymatic reactions for target detection (Fig. 1*B*; mode 2). ITP enables simultaneous target DNA recognition by Cas12–gRNA and the resulting target-activated cleavage of ssDNA reporters. This step is carried out at room temperature, and a fluorescence readout is used to detect the presence of preamplified nucleic acids (Fig. 1*B*). LAMP primers and gRNAs for SARS-CoV-2 detection used in this work were originally published and validated by Broughton et al. (3).

### ITP Preconcentrates and Cofocuses Cas12–gRNA, Target DNA, and Reporter ssDNA into a ∼100-pL Reaction Volume.

To test our hypothesis that Cas12–gRNA can be controlled and cofocused with other CRISPR reagents using an electric field gradient (in a device with no moving parts), we directly visualized the electrokinetic transport of Cas12–gRNA complex and reporter ssDNA in ITP. In these experiments, we used a Cy5-tagged gRNA and a green fluorescent ssDNA reporter (*SI Appendix*, Table S1), and we imaged the electromigration of the ITP peak near the LE–TE interface (Fig. 1*C* and Movie S1). The reporter ssDNA (green) and Cas12–gRNA complex (red) were found to electromigrate with the same velocity, as indicated by the slope (d*x*/d*t*) of the red and green fluorescence intensity fields (Fig. 1*C*). Further, a significant overlap of Cas12–gRNA and reporter ssDNA intensity profiles was observed experimentally (Fig. 1*C*, *Inset* and *SI Appendix*, Fig. S3), which indicates these molecules cofocus in ITP, therefore accelerating reaction. Additionally, we expect the unlabeled target DNA to also cofocus in the ITP peak, since its mobility is bracketed by the LE and TE (see *Materials and Methods*). Calibrated fluorescence measurements indicate that the concentration of Cas12–gRNA complex, target DNA, and ssDNA reporters increase within ∼100 pL of the ITP peak region by order ∼1,000-fold compared to the initial concentrations (see standard curve and fluorescence measurements during ITP in *SI Appendix*, Fig. S4, and refer to *Materials and Methods* for ITP peak volume estimation). Such preconcentration of nucleic acids in ITP has been previously reported (12, 13). The increased concentration of molecules within a tiny volume achieved using ITP dramatically speeds up the diffusion-limited enzymatic kinetics (5) of CRISPR-based detection assays.

### ITP–CRISPR-Based Assay for Rapid Detection of N and E Genes of Viral RNA and Human RNase P Gene.

**Fig. 2.**
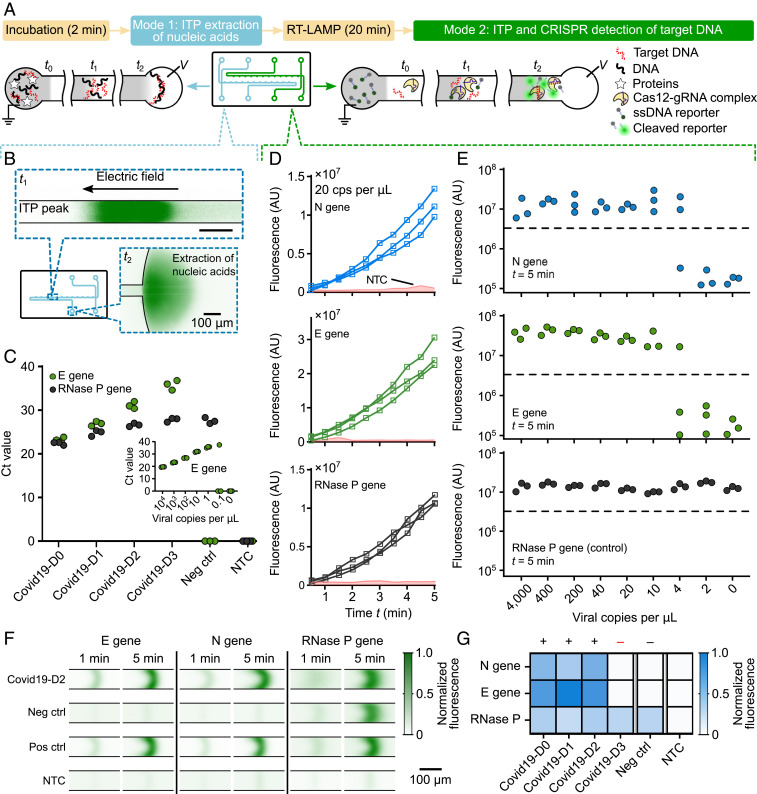
Demonstration of the assay using both contrived and clinical NP swab samples. (*A*) Schematic of ITP extraction and ITP–CRISPR detection operational modes. A 2-min preincubation at 62 °C in lysis buffer is performed prior to on-chip ITP extraction (mode 1). Twenty-minute LAMP at 62 °C is performed off-chip prior to on-chip ITP–CRISPR detection (mode 2). (*B*) Experimental images of on-chip labeled DNA and RNA focused (green) within the ITP peak during nucleic acid extraction from clinical NP sample (mode 1). Ten microliters of NP swab sample is used as input. Nucleic acids are transferred into the LE reservoir. (*C*) RT-PCR of E gene and RNase P gene on ITP-extracted nucleic acids from clinical NP samples. Covid19-D1 to Covid19-D3 are 1:10 serial dilutions of Covid19-D0 in negative control (see *Materials and Methods* for details about clinical samples preparation). *Inset* shows RT-PCR standard curve for the E gene. (*D*) Monitoring of fluorescence signal for contrived samples (mode 2). Fluorescence signal for LAMP amplicons of N gene, E gene, and RNase P targets versus time for a contrived sample containing pooled nucleic acid extract from negative clinical NP swabs spiked with 20 viral genomes per microliter of reaction (*n* = 3). Shaded region represents signal from no template control (NTC) (*n* = 10). (*E*) Analytical LOD of ITP–CRISPR method. Fluorescence readout at 5 min of ITP–CRISPR assay (mode 2). An end-point fluorescence threshold value of 3 × 10^6^ AU was used to determine the result. Synthetic SARS-CoV-2 RNA controls were spiked into pooled negative clinical NP swab extracts before LAMP. (*F*) Fluorescence visualization of ITP peak during ITP–CRISPR detection. The ssDNA reporters with quencher/fluorophore are cleaved by Cas12–gRNA on recognition of target DNA, resulting in an increased fluorescence. (*G*) Results of the complete 30-min assay on clinical NP swab samples. One of the positive samples (Covid19-D3) was verified to be below the 10 copies per microliter LOD of our assay.

We next developed a protocol for the detection of RT-LAMP−amplified complementary DNA (cDNA) of SARS-CoV-2 viral RNA using ITP-mediated CRISPR–Cas12 DNA detection. Upon CRISPR–Cas12 binding to the target cDNA of SARS-CoV-2 viral RNA targets, Cas12 promiscuously cleaves ssDNA (3, 5). The activated Cas12 cleaves reporter ssDNA probes labeled with a fluorophore−quencher pair, resulting in unquenching of the fluorophore and an increase in observed fluorescence. Thus, a positive detection occurs when the fluorescence of the ITP peak rapidly increases with time (and above a threshold; see *Materials and Methods*), while the result is negative when there is minimal change in fluorescence (Figs. 1*D* and 2*D* and *E* and Movies S2 and S3). We also evaluated the analytical limit of detection (LOD) of the ITP–CRISPR method (Fig. 2*E*). For this set of experiments, contrived samples were used which consisted of viral RNA spiked into pooled nucleic acid extracts from negative clinical NP swab samples. Fluorescence intensity of the moving ITP peak was measured for the N, E, and RNase P genes independently in separate reactions (Fig. 2*D* and *SI Appendix*, Figs. S5–S7), and the signal at 5 min was used as the endpoint readout. The LOD of the ITP–CRISPR method was found to be 10 copies per microliter of reaction, which is the same as a recent CRISPR-based SARS-CoV-2 assay (3). Further, in the case of positive detection, a fluorescence signal above the threshold value was typically observed within 3 min (*SI Appendix*, Fig. S8). These results are in contrast to the 1 copy per microliter of LOD obtained for the 2-h RT-PCR protocol of Corman et al. (1) (Fig. 2*C*, *Inset*) and that reported by the Stanford clinical virology laboratory’s SARS-CoV-2 RT-PCR test (14). Lastly, we verified that microfluidic ITP-CRISPR detection and the typical CRISPR-based (3) approaches gave the same positive/negative result when tested with the same LAMP preamplified DNA (*SI Appendix*, Fig. S9).

### ITP Enables Rapid Extraction of Total Nucleic Acids from Raw NP Swab Samples.

We also demonstrated on-chip ITP extraction of total nucleic acids from raw clinical positive and negative NP swab samples (Figs. 1*B* and 2 *A*, *B*, and *C*). To validate our extraction method, we performed RT-PCR for the E gene and RNase P control (Fig. 2*C*). The E gene assay, from WHO/Corman et al. (1), shows sensitivity similar to the CDC N2 target (2, 15) and is in use at the Stanford hospital clinical virology laboratory under emergency use authorization by the US Food and Drug Administration (14). We used this E gene assay to avoid any discrepancies due to the assay target. To enable systematic quantification (Fig. 2*C*), we used one positive clinical NP swab specimen (Covid19-D0) and diluted it 1:10, 1:100, and 1:1,000 (Covid19-D1 to Covid19-D3) in pooled negative clinical NP swab specimens in VTM. The pooled negative NP swab specimens were used as negative control (see *Materials and Methods*).

We observed that ITP-extracted nucleic acids showed E gene amplification on positive samples, while the RNase P reaction amplified across all patient samples (Fig. 2*C*). These results suggest that ITP-extracted nucleic acids are compatible with downstream amplification methods (such as PCR and LAMP) for SARS-CoV-2. Our results corroborate several previous studies that have shown compatibility of ITP with downstream amplification methods such as PCR, LAMP, and RPA. Examples include ITP-aided assays for the detection of urinary tract infections (9), malaria (16), *Listeria monocytogenes* (17), and pathogenic *Escherichia coli* (18), among many others.

We note here that Proteinase K is used in our assay for sample lysis even though it is an inhibitor of PCR, LAMP, and Cas12a. We prevent this inhibition by designing the pH of ITP buffers to be less than the pI (= 8.9) of Proteinase K (10, 11). This ensures Proteinase K is positively charged and electromigrates in the direction opposite to DNA and RNA. Likewise, other high-concentration cations such as Na^+^ and K^+^ present in the swab lysate [and which can inhibit LAMP and PCR (11, 19–21)] do not electromigrate in the direction of nucleic acids. Further, we use 20 mM Tris⋅HCl as the ITP extraction buffer (see *Materials and Methods*), since this low-concentration buffer is compatible with downstream PCR (22) and LAMP.

### Demonstration of the 30-Min Protocol on Clinical Samples.

Next, we combined off-chip RT-LAMP with on-chip ITP nucleic acid extraction and ITP–CRISPR detection methods and performed the complete 30-min assay (raw sample to result) on samples Covid19-D0 to Covid19-D3 (*SI Appendix*, Fig. S10). As noted earlier, samples Covid19-D1 to Covid19-D3 are 1:10 serial dilutions of Covid19-D0. A detectable fluorescence signal for the E gene and N gene targets was observed in three out of the four positive samples (Fig. 2*G*), while the pooled negative sample (negative control) showed a negative result (Fig. 2*F* and *G*). RNase P controls showed consistent positive signal in all samples. Using RT-PCR (Fig. 2*C*), we verified that the single undetected positive sample (Covid19-D3) was below the 10 copies per microliter of LOD of our ITP-CRISPR assay, but above the 1 copy per microliter of LOD of RT-PCR method (1, 14).

### Evaluation of ITP-Mediated CRISPR–Cas12 Detection on Clinical Samples.

**Fig. 3.**
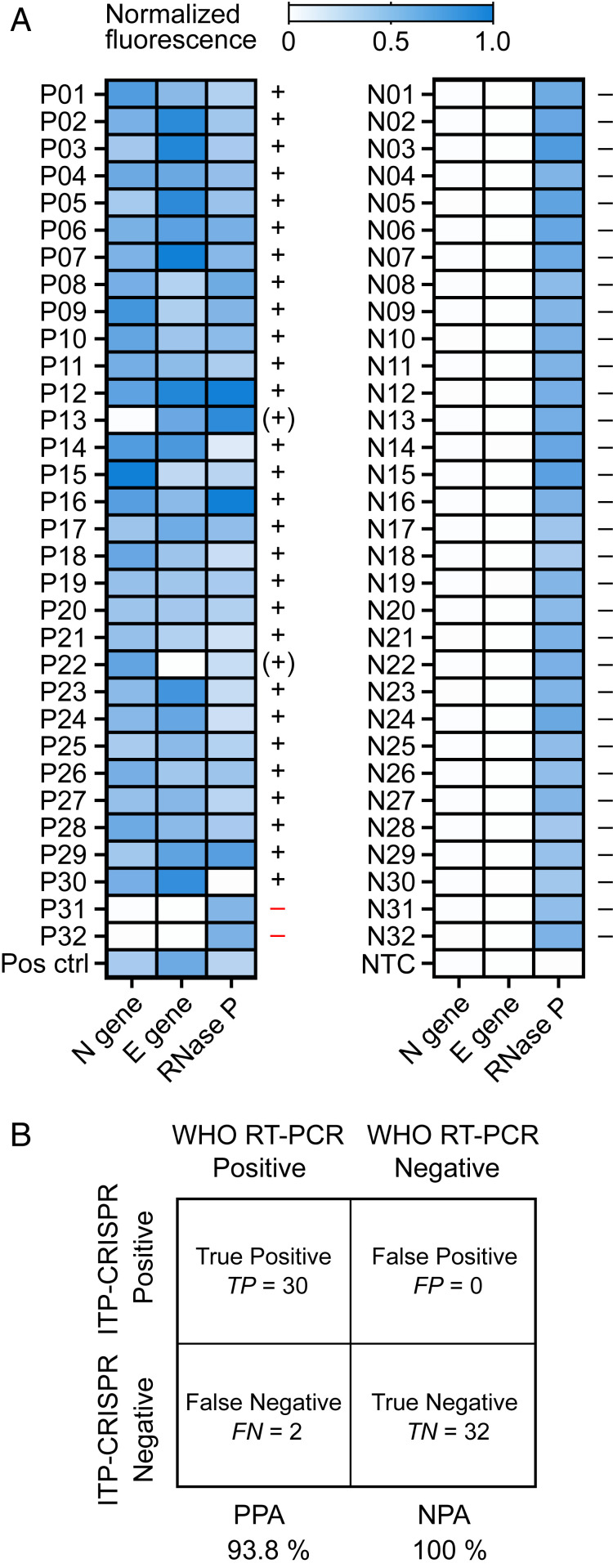
Evaluation of the ITP–CRISPR assay on clinical samples. (*A*) End-point fluorescence readouts of the ITP–CRISPR detection assay (mode 2) for the N, E, and RNase P genes performed on clinical samples. NP swab extracts from 32 positive (*Left*) and 32 negative (*Right*) patients, determined by the Stanford clinical virology laboratory’s SARS-CoV-2 RT-PCR assay (14), were tested. RT-LAMP was performed off-chip for 30 min prior to 5 min of on-chip ITP–CRISPR detection. Positive/negative test interpretation is indicated by +/− (*SI Appendix*, Table S2), and parentheses are used to indicate cases where only one of N gene or E gene was detected. (*B*) Summary of test results. ITP–CRISPR detection is compared against the Stanford hospital clinical laboratory RT-PCR assay (14) which is adapted from WHO/Corman et al. (1). The ITP–CRISPR assay showed 96.9% overall agreement with a kappa value of 0.94 (95% CI: 0.85 to 1.0). Kappa statistics were calculated using GraphPad software.

We evaluated the performance of ITP–CRISPR detection (mode 2) on a total of 64 patient specimens. Of these, 32 samples were positive and 32 were negative for SARS-CoV-2, as determined by the Stanford clinical virology laboratory’s RT-PCR assay (14). RT-LAMP was performed for 30 min on preextracted nucleic acids from patient NP swabs followed by 5 min of on-chip ITP–CRISPR detection (mode 2). We here chose to directly use NP swab extracts as a controlled evaluation of the performance of, specifically, the ITP-enhanced CRISPR–Cas12 reaction process and detection method. For this study, we performed RT-LAMP for 30 min (instead of 20 min) to improve sensitivity, as suggested by the work Broughton et al. (3). The ITP–CRISPR method correctly detected 30 out of 32 positive samples, and we observed no false positives on the 32 negative samples (Fig. 3*A*). Among the positive samples, 28 showed a positive signal for both the N and E genes, while two samples (P22 and P13) showed a positive signal for only one of N or E genes. We interpreted the test result as positive when at least one of N or E gene targets was detected (see *SI Appendix*, Table S2 for the ITP–CRISPR test interpretation methodology). RNase P control gene was detected in 63 out of 64 samples (all except P30). We found that our ITP–CRISPR method consistently and correctly detected positive samples when the Ct value [determined by the RT-PCR assay (14); *SI Appendix*, Table S3] was less than 33. This Ct value is consistent with our estimated LOD of 10 copies per microliter for the ITP–CRISPR method. The two undetected positive samples in Fig. 3 each had Ct values greater than 35, which was below the LOD of our assay. The positive (PPA) and negative (NPA) predictive agreements of the ITP–CRISPR detection method were 93.8% and 100%, respectively (Fig. 3*B*). Our microfluidic assay’s LOD, PPA, and NPA were comparable to the assay of Broughton et al. (3). However, in contrast to the work of Broughton et al. (3), we here demonstrate automated electric field extraction and selective focusing of target nucleic acids from raw samples, electric field control and enhancement (via preconcentration) of the various CRISPR reactions, and simultaneous electric field preconcentration of cleaved reporters for ease of fluorescence detection.

## Discussion

We developed an electrokinetic microfluidic method broadly applicable to CRISPR-based diagnostics. Our method involves ITP-based nucleic acid extraction from raw sample, isothermal reverse transcription and amplification, and then a CRISPR assay enhanced by ITP with a total assay time of around 30 min to 40 min (from raw sample to result). We applied our method to the rapid detection of SARS-CoV-2 RNA from both contrived and clinical NP swab samples and demonstrated clinically relevant diagnostic performance.

An advantage of our microfluidic ITP–CRISPR assay is the minimal consumption of reagents. For example, our assay uses less than 0.2 μL of reagents on chip for the CRISPR reactions, and these reagent volumes are more than 100 times lower than existing CRISPR assays (3, 4). We also integrated rapid, ITP-based on-chip nucleic acid extraction into the workflow. In this work, ITP was used to perform two important steps of our assay: automated nucleic acid extraction, and control and enhancement of CRISPR enzymatic reactions for detection. Note that this is in contrast with the CRISPR–Cas12 assay of Broughton et al. (3) which required front-end extraction and purification of nucleic acids from raw patient NP swab samples using standard sample preparation equipment. Interestingly, the extraction procedure used in their work requires several manual steps, takes up to 1 h, and uses bulky equipment (such as a centrifuge). A comparison of our SARS-CoV-2 RNA detection method with the CRISPR–Cas12 assay of Broughton et al. (3) and the WHO RT-PCR assay is provided in Table 1. Of course, all CRISPR-based assay technologies offer reconfigurability to the detection of novel pathogens by redesign of the preamplification primers and gRNAs (which are synthetic nucleic acid components). For the current ITP-based assay, this reconfiguration should not require any changes to the microfluidic chip design, buffers, or hardware.

**Table 1.** Comparison of ITP–CRISPR-based detection with the conventional CRISPR-based (3) and RT-PCR assays (1, 2)

	ITP–CRISPR assay (extraction/preamplification/CRISPR–Cas)	Conventional CRISPR assay (preamplification/CRISPR–Cas)	RT-PCR
Target genes	N gene, E gene	N gene, E gene	N gene, E gene
Control gene	RNase P	RNase P	RNase P
LOD	10 copies per μL	10 copies per μL	1 copy per μL
Requires separate nucleic acid extraction	No	Yes	Yes
Time for nucleic acid extraction (approximate)	5 min (on-chip)	30 min to 1 h (with bulky equipment)	30 min to 1 h (with bulky equipment)
Time for reaction (amplification+detection; approximate)	30 min to 35 min (high-temperature amplification, room temperature detection)	30 min to 40 min (high- temperature amplification and detection)	2 h (high- temperature amplification and detection)
Total assay time (raw sample to result)	30 min to 40 min	1 h to 1.5 h	2.5 h to 3 h
Assay control	Electric field; automated	Manual	Manual
Quantitative	No	No	Yes
Reagent consumption	<0.2 μL (on-chip)	Up to 100 μL (in tube)	20 μL (in tube)

A disadvantage of our current assay’s workflow is the requirement of intermediate off-chip manual steps for sample lysis and LAMP. Our assay is also currently limited to processing 10 μL of raw sample as input due to constraints placed by the microfluidic chip design, and this could affect sensitivity. Scaled-up ITP channels for extraction may mitigate the latter limitation. For example, a recently developed commercial device (IONIC ITP system, Purigen Biosystems, Inc.) uses ITP for nucleic acid extraction with input sample volumes of 200 μL. Although our work demonstrated the ITP–CRISPR assay using laboratory-scale equipment (such as microscope, sourcemeter, and camera), ITP-based detection systems can be miniaturized into hand-held portable devices with fully integrated electronic and optical hardware components. For example, a portable ITP system developed by Kaigala et al. (23) integrated into a single device a miniature laser (for laser-induced fluorescence), optical filters, photodiode sensor, a 300-V-output DC-to-DC converter complementary metal–oxide–semiconductor (CMOS) chip powered by a 5-V universal serial bus (USB), and a microprocessor to capture and transmit detection signal via USB. Lastly, large-scale manufacturing of plastic microfluidic chips using injection molding would presumably significantly lower the cost of a (consumable) microfluidic chip per test.

There is growing demand for the development of rapid and sensitive field-deployable tests for nucleic acids, especially for use during pandemics such as COVID-19. Such tests can minimize turnaround times (currently, mean of ∼14 h for Stanford hospital COVID-19 test) between sample collection and result, alleviate the workload on centralized testing laboratories, and enable rapid actionable decisions for treatment and control of disease spread. Future work could include integration of our assay steps on a single microfluidic device and a portable readout system (e.g., *SI Appendix*, Fig. S11) to enable the development of an automated microfluidic platform for rapid ITP–CRISPR-based nucleic acid tests applicable at the point of care, including in low-resource settings.

## Materials and Methods

This study was conducted with the approval of the Stanford University Institutional Review Board (IRB protocol #48973), and individual consent was waived.

### Nucleic Acid Preparation.

Synthetic ssRNA control for SARS-CoV-2 variant (GenBank ID: MT007544.1) was obtained (Twist Biosciences) at a concentration of 1 million copies per microliter. The ssRNA control sequences were generated by the transcription of six nonoverlapping 5-kb gene fragments of SARS-CoV-2, providing greater than 99.9% coverage of the viral genome. For analytical LOD assays, dilutions of RNA stock solution were prepared in RNA reconstitution buffer (GeneLink). LAMP primers and gRNA targeting the N and E genes of SARS-CoV-2 and RNase P gene of human DNA were originally published by Broughton et al. (3), and the sequences are listed in *SI Appendix*, Table S1. LAMP primers (Elim Biosciences) were reconstituted in nuclease-free water, and gRNAs (IDT) were reconstituted in RNA reconstitution buffer.

For ITP cofocusing visualization experiments, the Mtb target DNA sequence was used (*SI Appendix*, Table S1). One micromolar stock solution of Mtb dsDNA was prepared by prehybridizing complementary ssDNA templates (Elim Biosciences) in a buffer containing 50 mM Tris⋅HCl, 5 mM MgCl_2_, and 1 mM (ethylenedinitrilo)tetraacetic acid at 37 °C. We designed a Cy5-labeled gRNA (IDT; *SI Appendix*, Table S1) to target the Mtb dsDNA sequence.

### Preparation of Contrived Samples for ITP–CRISPR Detection Assay.

Deidentified residual eluates from 40 negative NP swab samples were acquired from the Stanford clinical virology laboratory. Total nucleic acids were extracted from 500 µL of NP swab specimen using QIAsymphony DSP Virus/Pathogen Midi Kit and were eluted into 60 μL. The 40 eluates were pooled to provide human nucleic acid background to match the clinical specimens. The negative attribution of the aforementioned samples was based on the results of the Stanford SARS-CoV-2 RT-PCR test (14). Briefly, the protocol specifically targeted the E gene of SARS-CoV-2 and also tested for cross-reactivity among other high-priority pathogens from the same genetic family (including seasonal human coronaviruses) and among other pathogens likely to be present in the circulating areas. See protocol in ref. 14 for more details. Synthetic SARS-CoV-2 RNAs of known concentrations were combined with the pooled clinical nucleic acid extracts before performing analytical LOD experiments for the ITP–CRISPR assay.

### Microfluidic Chip and Preparation.

ITP-based nucleic acid extraction and ITP–CRISPR detection were performed using off-the-shelf glass microfluidic chips (model NS12AZ, Caliper Life Sciences—subsidiary of PerkinElmer, Inc.). A single chip consists of two cross-geometry channels wet-etched to a 20-μm depth with a 50-μm mask width, resulting in a channel width of 90 μm and a roughly D-shaped cross-section (*SI Appendix*, Fig. S14; see also ref. 24). The cross-sectional area of the channel is 1,628 μm^2^. The main channel length between the positive/negative electrodes is 72 mm (*SI Appendix*, Fig. S14). To avoid cross-contamination, ensure run-to-run repeatability, and provide uniform surface properties, the channels were rinsed in the following order before each ITP experiment: 10% bleach for 2 min, deionized (DI) water for 2 min, 1% Triton-X for 2 min, DI water for 2 min, 1 M NaOH for 2 min, and DI water for 2 min. Between each rinse step, the channel was completely dried using vacuum. For this study, a single chip was used for all experiments, and the aforementioned wash steps ensured no cross-contamination between samples. The buffer loading procedure and buffer placement in the channel sections are detailed in *SI Appendix*, Fig. S12.

### ITP Extraction of Total Nucleic Acids.

ITP was used to extract total nucleic acids from 10 μL of primary NP swab clinical samples in VTM. Samples were acquired from the Stanford clinical virology laboratory. Ten microliters of NP sample was mixed with 1.1 μL of 10× lysis buffer and incubated at 62 °C for 2 min. The 1× composition of lysis buffer included 1.5% Triton X, 1 mg/mL Proteinase K, and 0.1 mg/mL carrier RNA (Thermo Fisher). Following incubation, 1 μL of 300 mM Hepes buffer was added, and 10 μL of this mixture was dispensed in the TE reservoir on-chip (*SI Appendix*, Fig. S12). The LE buffer in the main channel consisted of 100 mM Tris⋅HCl (pH 7.5), 1 U/μL RNasin Plus, 0.2% Triton X, 1% of 1.3-MDa Polyvinylpyrrolidone (PVP) and 1× SYBR Green I. SYBR Green I was used to visualize the ITP peak which contained nucleic acids (Fig. 2*B*). The 10-μL extraction buffer in the LE reservoir consisted of 20 mM Tris⋅HCl (pH 7.5), 1 U/μL RNasin Plus, and 0.1 mg/mL carrier RNA. The low-concentration extraction buffer does not significantly modify the LAMP/PCR master mix buffer composition, and was thus used to ensure compatibility with downstream PCR and LAMP amplification (22). The effective electrophoretic mobilities of chloride (LE coion) and Hepes (TE coion) are 7.91 × 10^−8^ m^2^⋅V^−1^⋅s^−1^ and 2.09 × 10^−8^ m^2^⋅V^−1^⋅s^−1^, respectively. The free solution mobility of nucleic acids is buffer dependent and only a weak function of sequence length, and the value is typically between 3 × 10^−8^ m^2^⋅V^−1^⋅s^−1^ and 4 × 10^−8^ m^2^⋅V^−1^⋅s^−1^ (25). Importantly, the free solution mobility of nucleic acids is bracketed between our chosen LE and TE coions (10, 26). ITP extraction of nucleic acids was performed at constant voltage of 1 kV supplied by a Keithley 2410 high-voltage sourcemeter (*SI Appendix*, Figs. S12 and S13).

### RT-LAMP Reactions.

RT-LAMP reactions were carried out off-chip (in tubes) with the WarmStart LAMP Kit (NEB) using the manufacturer’s recommended protocol. The final concentrations of LAMP primers were 1.6 μM for forward inner primer (FIP) and backward inner primer (BIP), 0.2 μM for forward outer primer (F3) and backward outer primer (B3), and 0.8 μM for forward loop primer (LF) and backward loop primer (LB), as used in Broughton et al. (3). Reactions were performed with a final volume of 10 μL, and were set up separately for N, E, and RNase P genes. LAMP reaction mixtures were incubated at 62 °C for 20 min.

For ITP–CRISPR analytical LOD experiments (Fig. 2*E*), 4 μL of contrived sample containing a mixture of viral RNA (2 μL) and pooled negative clinical NP swab extracts (2 μL) was used as template. For tests involving the complete 30-min assay on clinical patient samples (Fig. 2 *F* and *G*), we used 3 μL of ITP-extracted nucleic acids as template for each LAMP reaction. For experiments that evaluated ITP–CRISPR detection on 64 clinical samples (Fig. 3), 4 μL of NP swab extracts was used as template, and RT-LAMP was carried out off-chip for 30 min followed by 5 min of on-chip ITP–CRISPR detection (mode 2).

### Cas12–gRNA Complex Preparation.

A 10× Cas12–gRNA complex mixture was prepared by preincubating 1 μM of LbCas12a (NEB) with 1.25 μM gRNA in 1× NEBuffer 2.1 at 37 °C for 30 min. Cas12–gRNA complexes were prepared independently for N, E, and RNase P genes. For ITP cofocusing visualization experiment in Fig. 1*C*, a 10× Cas12–gRNA complex was prepared using 1 μM of LbCas12a (NEB) and 0.5 μM of Cy5-labeled gRNA. Here, a molar excess of LbCas12a was used to minimize free, unbound gRNA.

### ITP–CRISPR Detection.

The LE buffer consisted of 200 mM Tris, 100 mM HCl, 10 mM MgCl_2_, and 0.1% PVP. The TE buffer consisted of 100 mM Tris, 50 mM Hepes, 10 mM MgCl2, 0.1% PVP, and 250 nM ssDNA fluorescence quencher reporter (/56-FAM/TTATT/3IABkFQ/, IDT). Before each SARS-CoV-2 ITP–CRISPR detection experiment, 2 μL of the 10× LbCas12-gRNA complex was combined with 2 μL of the corresponding LAMP amplicon and 16 μL of LE buffer. For ITP cofocusing visualization experiments in Fig. 1*C*, 2 μL of the 10× LbCas12–gRNA complex was combined with 2 μL of preprepared Mtb dsDNA template and 16 μL of LE buffer. The on-chip buffer loading procedure is described in *SI Appendix*, Fig. S12.

The ITP–CRISPR detection experiments were performed at constant current of 4 μA supplied by a Keithley 2410 sourcemeter (*SI Appendix*, Figs. S12 and S13). Fluorescence images of the moving ITP peak were acquired in 30-s intervals using a CMOS camera (Hamamatsu ORCA-Flash4.0) mounted on an inverted epifluorescence microscope (Nikon Eclipse TE200). For widefield images of ITP peak in Fig. 1*B*, we used a microscope objective (Nikon) with 2× magnification and 0.1 NA objective to enable imaging over a wide field of view. For all other quantitative fluorescence measurements of the ITP peak, a 10× magnification and 0.4 NA (Nikon) objective was used.

For ITP cofocusing visualization experiments of Fig. 1*C*, a white LED (Thorlabs) excitation source was used to enable simultaneous imaging of a Cy5-labeled (red channel) gRNA of the Cas12-gRNA complex and cleaved FAM-labeled (green channel) ssDNA reporter molecules. During the experiment, we manually switched between filter cubes for the green and far-red emission wavelengths. For all other experiments involving ITP-based nucleic acid extraction and ITP–CRISPR assay quantification, we used blue LED excitation source with a green emission filter cube. Fluorescence measurements (Figs. 1 *C* and *D* and 2*F* and *SI Appendix*, Fig. S3) showed an ITP peak width of ∼40 μm to 70 μm across experiments. Using the channel cross-sectional area of 1,628 μm^2^, we estimate the volume of the ITP peak region to be of order ∼100 pL.

### Image Analysis of Fluorescence Readouts.

The fluorescence signal was calculated from raw experimental images using ImageJ software (NIH). Fluorescence intensity values were integrated over a predefined square region around the ITP peak. The dimension of the square region was around four channel widths. A background value was obtained by integrating the signal over a square region with the same dimensions in the same image and in a region significantly away from the ITP peak. The reported signal is the background subtracted integrated fluorescence intensity. For test interpretation, a threshold for end-point fluorescence signal was chosen to be a value that was fivefold greater than the signal from no template control. An end-point fluorescence signal above and below the threshold was interpreted as positive and negative detection, respectively.

### SARS-CoV-2 RT-PCR Assay.

The RT-PCR assay was performed using the ABI 7500 Fast DX (Applied Biosystems) instrument. We performed assays for the E and RNase P genes separately in 20-µL reaction volumes using the Luna Universal Probe One-Step RT-PCR Kit (New England Biolabs). The final concentrations of primer and probe were 400 and 200 nM, respectively. We followed the recommended protocols in Corman et al. (1) and the Stanford clinical virology laboratory’s SARS-CoV-2 RT-PCR test (14). For quantification from clinical samples, we used 8 and 2 µL of ITP-extracted nucleic acids for the E gene and RNase P gene reactions, respectively. For the E gene standard curve, we used 5 µL of various dilutions of synthetic RNA controls as template.

### Human Clinical Sample Collection and Preparation.

Clinical positive and pooled negative SARS-CoV-2 NP swab samples in VTM were collected at the Stanford Clinical Virology laboratory. For quantification experiments (Fig. 2 *C*, *F*, and *G*), the original positive clinical NP swab specimen (Covid19-D0) was diluted 1:10 (Covid19-D1), 1:100 (Covid19-D2), and 1:1,000 (Covid19-D3) in VTM from pooled negative clinical NP swab specimens. The sample label “D” indicates the log10 amount of dilution of the original positive sample. We used these serial dilutions to enable systematic quantification of extraction using RT-PCR and to estimate LOD of the ITP–CRISPR enzymatic assay on clinically representative samples. The negative control comprised of pooled negative clinical NP swab samples.

For evaluation of the ITP–CRISPR detection method on 32 positive and 32 negative clinical specimens (Fig. 3), total nucleic acids were extracted from 500-µL NP swab specimens using the QIAsymphony DSP Virus/Pathogen Midi Kit and eluted in 60 μL. Residual eluates after the clinical testing were deidentified and used for this study.

## Supplementary Material

Supplementary File

Supplementary File

Supplementary File

Supplementary File

## Data Availability

All study data are included in the article and *SI Appendix*.

## References

[1] Corman V. M., Detection of 2019 novel coronavirus (2019-nCoV) by real-time RT-PCR. Euro Surveill. 25, 2000045 (2020). 10.2807/1560-7917.ES.2020.25.3.2000045 PMC6988269 31992387

[2] Centers for Disease Control and Prevention, “2019-novel coronavirus (2019-nCoV) real-time RT-PCR diagnostic panel” (Catalog #2019-nCoVEUA-01, Centers for Disease Control and Prevention, 2020).

[3] Broughton J. P., CRISPR-Cas12-based detection of SARS-CoV-2. Nat. Biotechnol. 38, 870–874 (2020). 32300245 10.1038/s41587-020-0513-4 PMC9107629

[4] Joung J., Point-of-care testing for COVID-19 using SHERLOCK diagnostics. medRxiv:10.1101/2020.05.04.20091231 (8 May 2020).

[5] Chen J. S., CRISPR-Cas12a target binding unleashes indiscriminate single-stranded DNase activity. Science 360, 436–439 (2018). 29449511 10.1126/science.aar6245 PMC6628903

[6] Gootenberg J. S., Nucleic acid detection with CRISPR-Cas13a/C2c2. Science 356, 438–442 (2017). 28408723 10.1126/science.aam9321 PMC5526198

[7] Eid C., Santiago J. G., Isotachophoresis applied to biomolecular reactions. Lab Chip 18, 11–26 (2018). 10.1039/c7lc00852j 29018854

[8] Ackerman C. M., Massively multiplexed nucleic acid detection with Cas13. Nature 582, 277–282 (2020). 32349121 10.1038/s41586-020-2279-8 PMC7332423

[9] Bercovici M., Rapid detection of urinary tract infections using isotachophoresis and molecular beacons. Anal. Chem. 83, 4110–4117 (2011). 21545089 10.1021/ac200253x PMC3116659

[10] Persat A., Marshall L. A., Santiago J. G., Purification of nucleic acids from whole blood using isotachophoresis. Anal. Chem. 81, 9507–9511 (2009). 19831356 10.1021/ac901965v

[11] Rogacs A., Marshall L. A., Santiago J. G., Purification of nucleic acids using isotachophoresis. J. Chromatogr. A 1335, 105–120 (2014). 24444800 10.1016/j.chroma.2013.12.027

[12] Bercovici M., Han C. M., Liao J. C., Santiago J. G., Rapid hybridization of nucleic acids using isotachophoresis. Proc. Natl. Acad. Sci. U.S.A. 109, 11127–11132 (2012). 22733732 10.1073/pnas.1205004109 PMC3396536

[13] Persat A., Santiago J. G., MicroRNA profiling by simultaneous selective isotachophoresis and hybridization with molecular beacons. Anal. Chem. 83, 2310–2316 (2011). 21329391 10.1021/ac103225c

[14] FDA, Accelerated Emergency Use Authorization (EUA) summary SARS-CoV2 RT-PCR assay (Stanford Health Care Clinical Virology Laboratory). https://www.fda.gov/media/136818/download. Accessed 29 October 2020.

[15] Waggoner J. J., Triplex real-time RT-PCR for severe acute respiratory syndrome coronavirus 2. Emerg. Infect. Dis. 26, 1633–1635 (2020). 32294051 10.3201/eid2607.201285 PMC7323516

[16] Marshall L. A., Han C. M., Santiago J. G., Extraction of DNA from malaria-infected erythrocytes using isotachophoresis. Anal. Chem. 83, 9715–9718 (2011). 22074444 10.1021/ac202567j

[17] Eid C., Santiago J. G., Assay for *Listeria monocytogenes* cells in whole blood using isotachophoresis and recombinase polymerase amplification. Analyst (Lond.) 142, 48–54 (2017). 10.1039/c6an02119k 27904893

[18] Borysiak M. D., Kimura K. W., Posner J. D., NAIL: Nucleic Acid detection using Isotachophoresis and Loop-mediated isothermal amplification. Lab Chip 15, 1697–1707 (2015). 25666345 10.1039/c4lc01479k

[19] Schrader C., Schielke A., Ellerbroek L., Johne R., PCR inhibitors—Occurrence, properties and removal. J. Appl. Microbiol. 113, 1014–1026 (2012). 22747964 10.1111/j.1365-2672.2012.05384.x

[20] Kaneko H., Kawana T., Fukushima E., Suzutani T., Tolerance of loop-mediated isothermal amplification to a culture medium and biological substances. J. Biochem. Biophys. Methods 70, 499–501 (2007). 17011631 10.1016/j.jbbm.2006.08.008

[21] Francois P., Robustness of a loop-mediated isothermal amplification reaction for diagnostic applications. FEMS Immunol. Med. Microbiol. 62, 41–48 (2011). 21276085 10.1111/j.1574-695X.2011.00785.x

[22] Rogacs A., Qu Y., Santiago J. G., Bacterial RNA extraction and purification from whole human blood using isotachophoresis. Anal. Chem. 84, 5858–5863 (2012). 22816776 10.1021/ac301021d

[23] Kaigala G. V., Miniaturized system for isotachophoresis assays. Lab Chip 10, 2242–2250 (2010). 20571691 10.1039/c004120c

[24] Shkolnikov V., Santiago J. G., A method for non-invasive full-field imaging and quantification of chemical species. Lab Chip 13, 1632–1643 (2013). 23463253 10.1039/c3lc41293h

[25] Stellwagen N. C., Gelfi C., Righetti P. G., The free solution mobility of DMA. Biopolym. 42, 687–703 (1997). 10.1002/(SICI)1097-0282(199711)42:6<687::AID-BIP7>3.0.CO;2-Q 9358733

[26] Garcia-Schwarz G., Rogacs A., Bahga S. S., Santiago J. G., On-chip isotachophoresis for separation of ions and purification of nucleic acids. J. Vis. Exp. e3890 (2012). 22415002 10.3791/3890 PMC3399465

