# Supplementary material for: Electric field-driven microfluidics for rapid CRISPR-based diagnostics and its application to detection of SARS-CoV-2
Source: Proc Natl Acad Sci U S A. 2020 Nov 4;117(47):29518–25. doi: 10.1073/pnas.2010254117 (PMC7703567; doi:10.1073/pnas.2010254117)
Supplement: Supplementary File [file pnas.2010254117.sapp.pdf]

Supplementary Information for

Electric-field-driven microfluidics for rapid CRISPR-based diagnostics and its application to detection of SARS-CoV-2

Ashwin Ramachandran[1], Diego A. Huyke[2], Eesha Sharma[3], Malaya K. Sahoo[4], ChunHong Huang[4], Niaz Banaei[4,5], Benjamin A. Pinsky[4,5], Juan G. Santiago[2,*]

[1] Department of Aeronautics & Astronautics, Stanford University, California, USA 94305
[2] Department of Mechanical Engineering, Stanford University, California, USA 94305
[3] Department of Biochemistry, Stanford University, Stanford, California, USA 94305
[4] Department of Clinical Pathology, Stanford University, Stanford, California, USA 94305
[5] Department of Medicine, Division of Infectious Diseases and Geographic Medicine, Stanford University, Stanford, California, USA 94305

* Corresponding author: Juan G. Santiago, Department of Mechanical Engineering, Stanford University, Stanford, CA 94305, Office (650)-736-1283.
Email: juan.santiago@stanford.edu

This PDF file includes:

Figures S1 to S14
Tables S1 to S3
Legends for Movies S1 to S3
SI References

Other supplementary materials include the following:

Movies S1 to S3

Supplementary Information Text

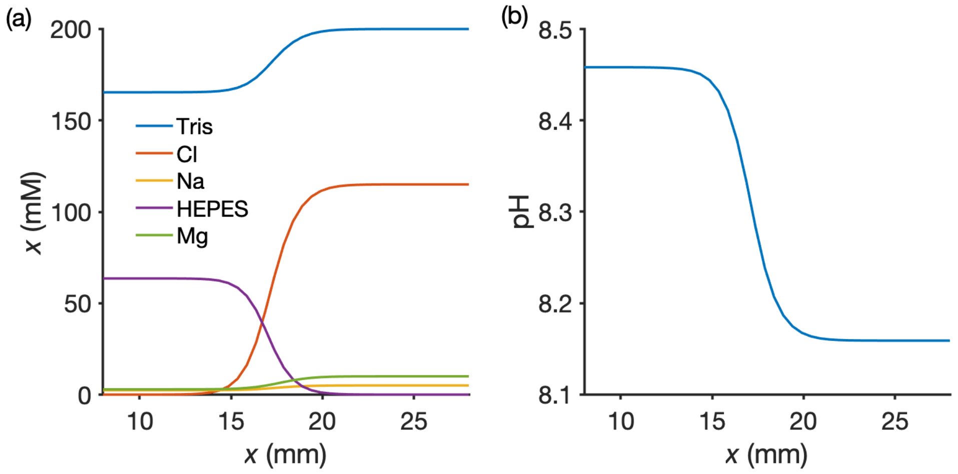

Figure S1. Simulation of ITP-CRISPR detection using Spresso (1)**.** (a) Concentration of ions versus *x* near the LE-TE interface. Applied electric field is in the direction from right to left. Not shown is the concentration of analyte ions (focused at the LE-TE interface region) since their concentrations are several orders of magnitude smaller than the buffer ions. (b) pH versus *x* near the ITP peak region. Note that the pH in the LE-TE interface varies between 8.16 (in the LE region) and 8.46 (in the adjusted TE region).

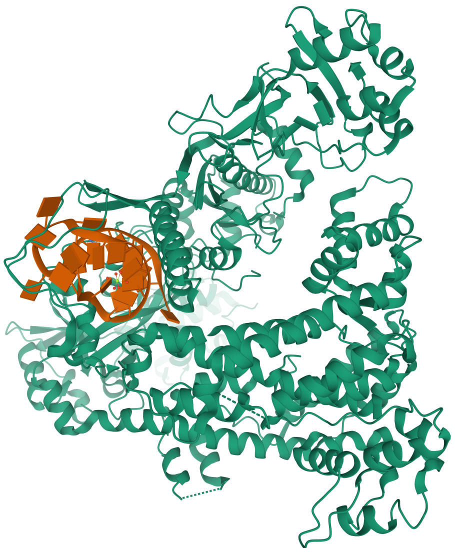

Figure S2. Structure of Cpf1(Cas12a)/RNA complex generated using PyMOL (PDB: 5id6) and calculation of theoretical isoelectric point. The source organism is wild-type *Lachnospiraceae bacterium* ND2006 (same as that of EnGen LbaCas12a, NEB). The theoretical isoelectric point (pI) computed using the ExPASy tool (compute pI/Mw) for this entire LbaCas12a protein sequence (UniProtKB: A0A182DWE3) is 8.39. When Cas12 (shown in green) complexed with RNA (shown in orange) is present in a buffer of pH around ~8.3 (between LE and TE), we hypothesize that the combined Cas12a-gRNA complex has a net negative charge in solution. Further, based on experiments and simulations, we estimate the effective electrophoretic mobility of Cas12a-gRNA complex is between 2.09 x 10^{-8} m^2/V-s (HEPES) and 7.91 x 10^{-8} m^2/V-s (Chloride) for our ITP chemistry.

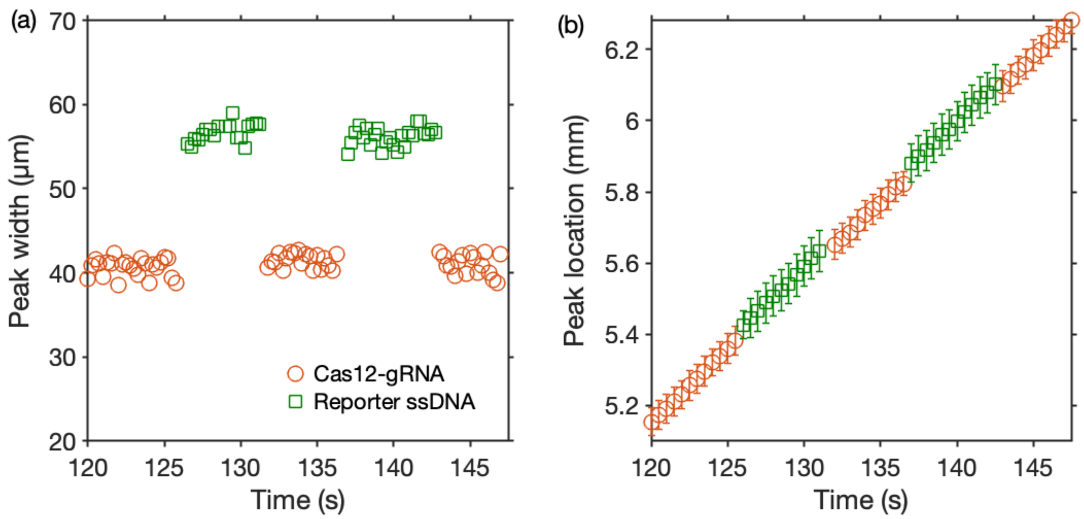

Figure S3. ITP peak width and location for Cas12-gRNA and ssDNA reporters when co-focused in ITP. (a) ITP peak width estimated as the full width at half maximum of the fluorescence imaging data, and (b) ITP peak location, versus time for the Cas12-gRNA complex (circle symbol) and reporter ssDNA (square symbol). Peak locations were determined by fitting an axial Gaussian distribution to the peak image data and determining the center of the Gaussian peaks. Uncertainty bars indicate peak location ± one peak width. Data presented here corresponds to the spatiotemporal intensities in Fig. 1c. Peak width of the reporter ssDNA (~58 µm) is greater than that of Cas12-gRNA complex (~40 µm), and the peak location of ssDNA is slightly ahead (~ 20 µm) of Cas12-gRNA. However, Cas12-gRNA and ssDNA concentration profiles significantly overlap as can be seen from the spatial profiles of fluorescence intensities in Fig. 1c inset. This overlap and preconcentration results in rapid Cas12 enzymatic activity on reporter molecules.

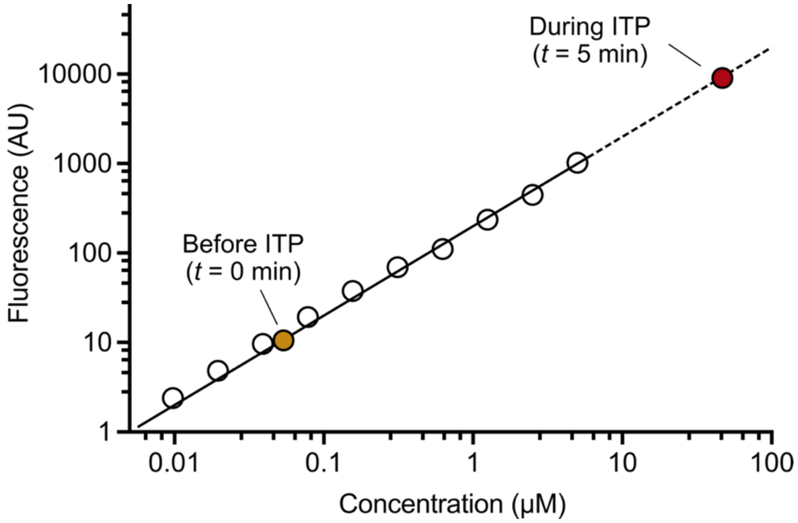

Figure S4. Fluorescence calibration curve. Various concentrations of Cy5-tagged gRNA (open symbols) were used to generate a fluorescence signal calibration curve. ITP preconcentrates Cas12-gRNA, reporters, and target DNA by order ~1000-fold compared to initial loading concentrations. All these molecules are concentrated from ~0.1 μL initial volume (on-chip, before ITP) to a ~100 pL volume (on-chip, during ITP). The increase in concentration of enzyme and substrate significantly accelerate production rates.

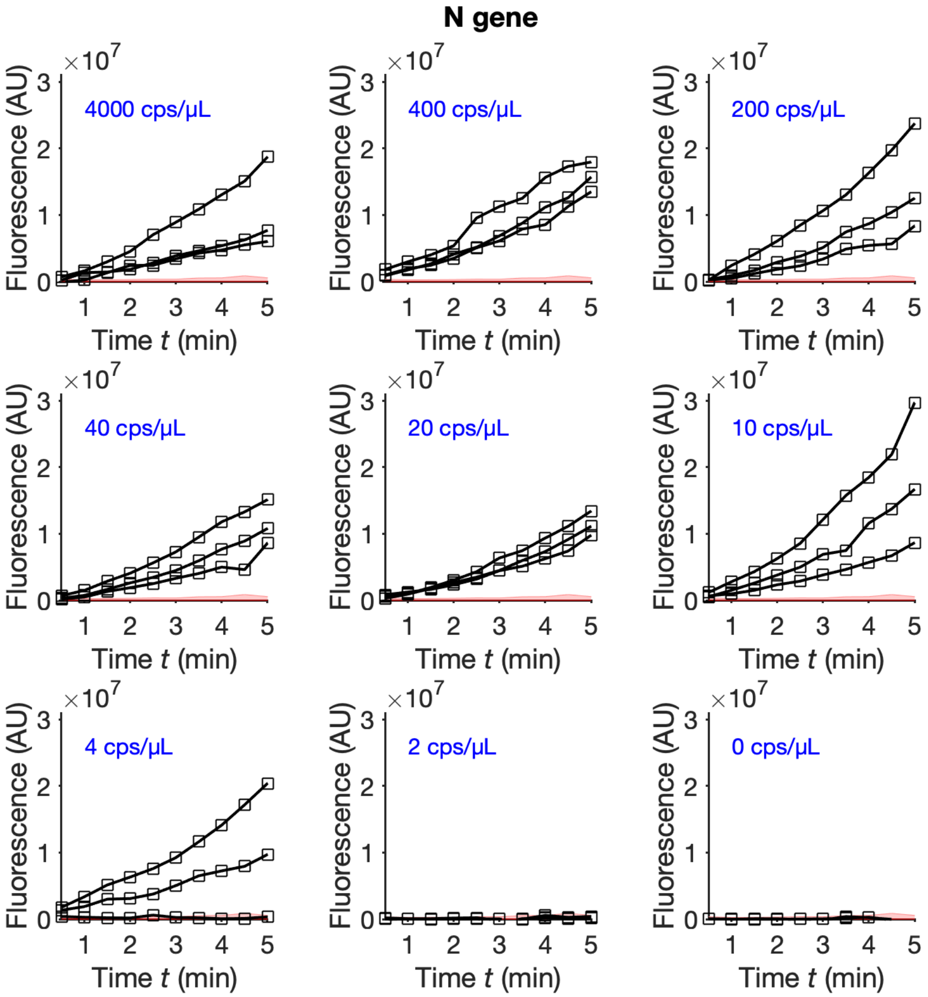

Figure S5. Experimental measurements of fluorescence signal for ITP-CRISPR detection of N gene in contrived samples. Area-integrated image fluorescence intensity (in the microfluidic channel) versus time during N gene ITP-CRISPR detection on contrived samples. Data was recorded every 30 s. Three replicates are shown for each concentration. The red shaded region shows signal from no template control.

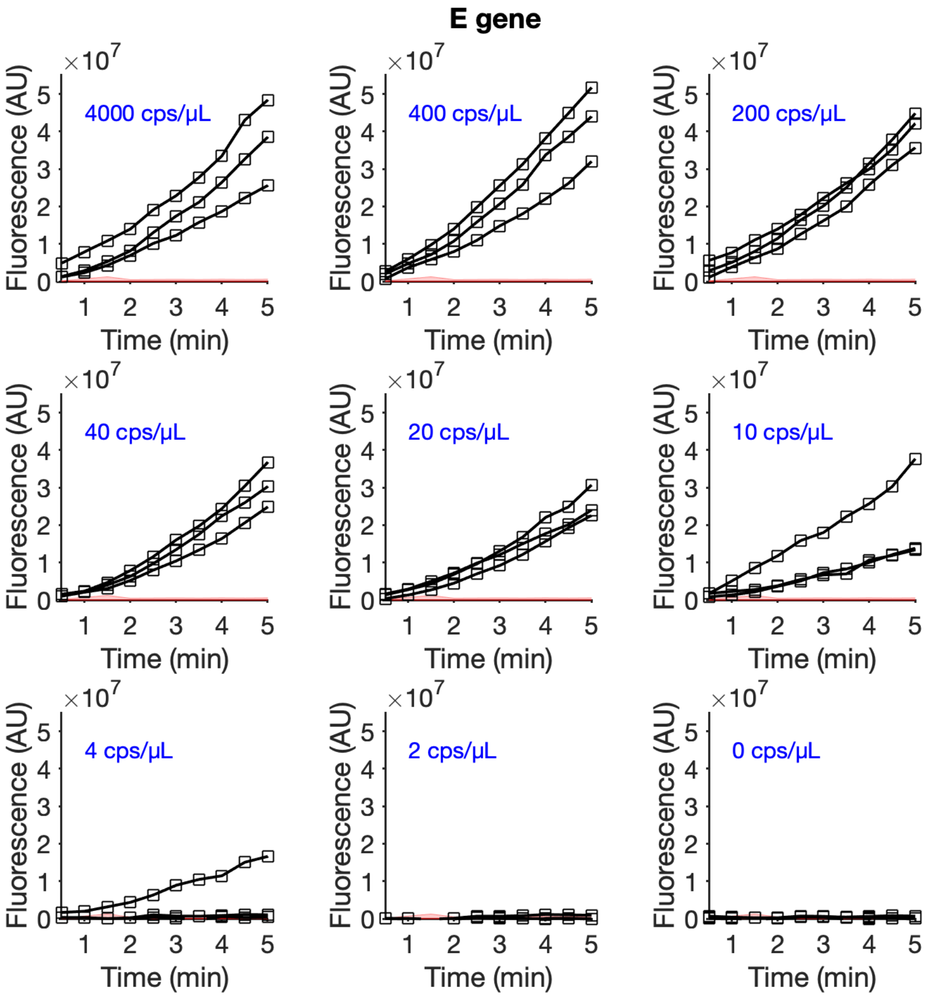

Figure S6. Experimental measurements of fluorescence signal for ITP-CRISPR detection of E gene in contrived samples. Area-integrated image fluorescence intensity (in the microfluidic channel) versus time during E gene ITP-CRISPR detection on contrived samples. Data was recorded every 30 s. Three replicates are shown for each concentration. The red shaded region shows signal from no template control.

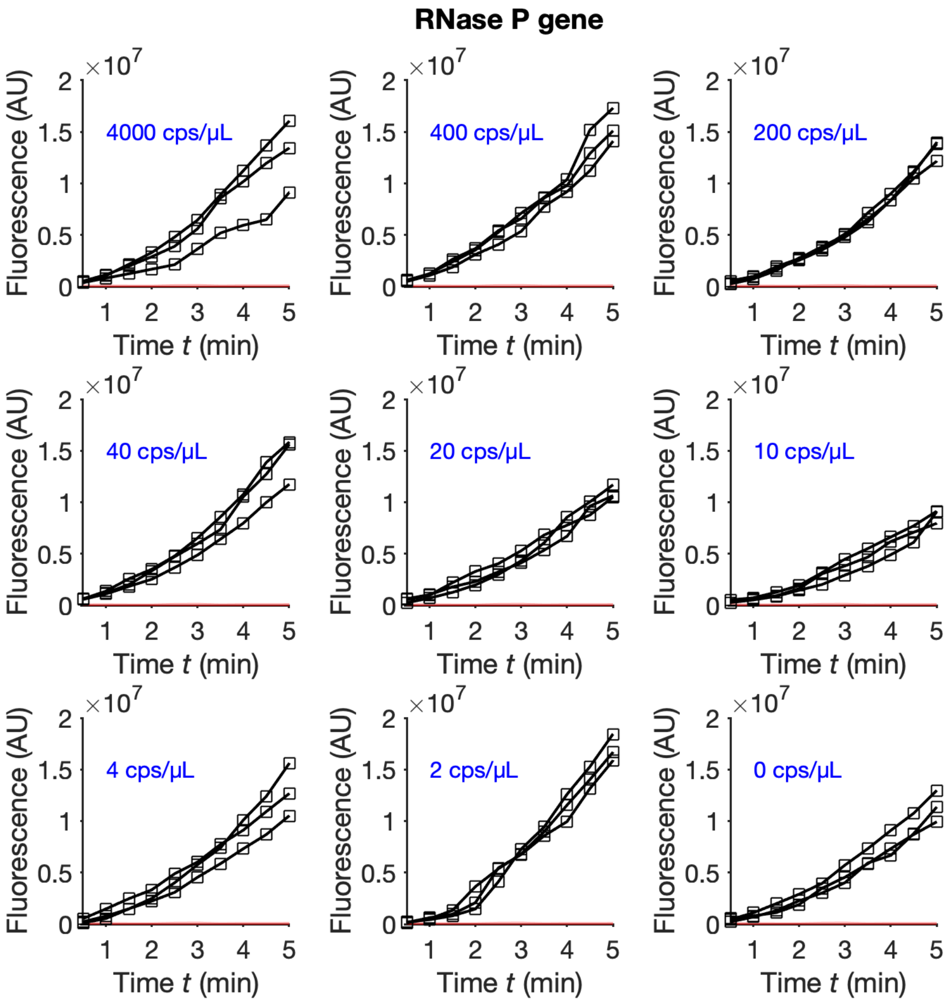

Figure S7. Experimental measurements of fluorescence signal for ITP-CRISPR detection of RNase P gene in contrived samples. Area-integrated image fluorescence intensity (in the microfluidic channel) versus time during RNase P gene ITP-CRISPR detection on contrived samples. Data was recorded every 30 s. Three replicates are shown for each concentration. The red shaded region shows signal from no template control. Note that human DNA from pooled negative nasopharyngeal swab extracts was present as background in all contrived samples.

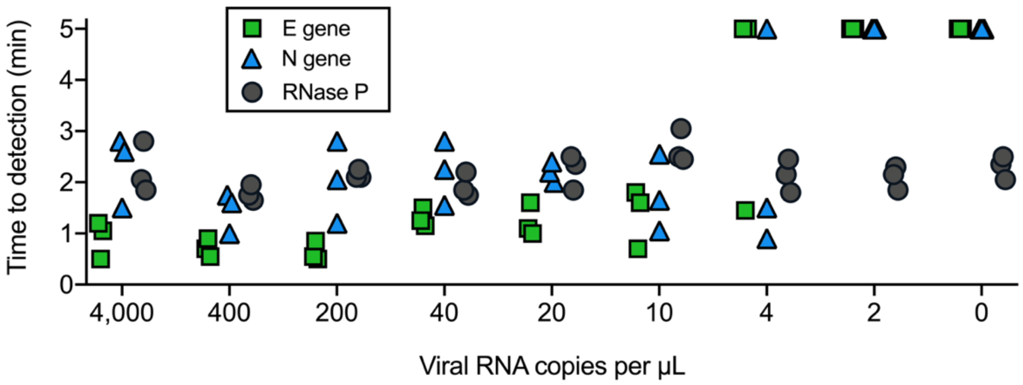

Figure S8. Time to signal detection on contrived samples for the ITP-CRISPR assay. A positive detection occurs when the measured fluorescence signal crosses a threshold value (here, equal to $3x10^6$ AU; data from Figs. 2e, S5, S6, S7). The time to signal detection is the time at which the measured signal crossed the threshold value. Samples for which signal did not exceed the threshold after 5 min of assay time are denoted by symbols at 5 min.

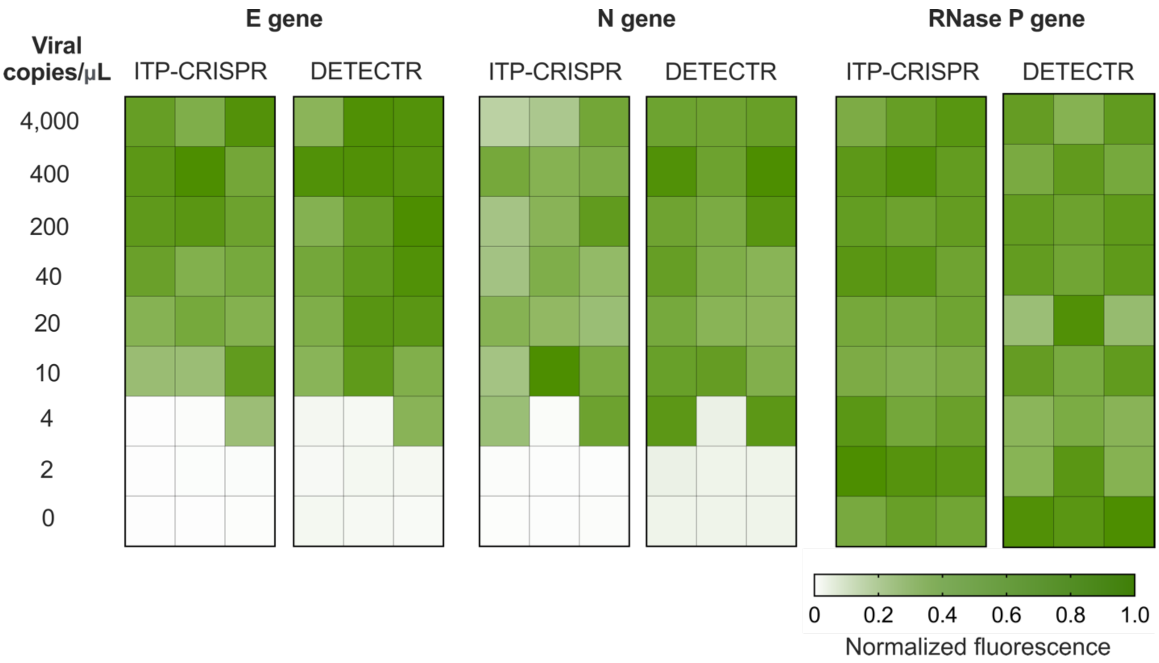

Figure S9. Comparison of ITP-CRISPR and DETECTR-based assays for SARS-CoV-2 detection on contrived samples. Shown are normalized fluorescence values for three replicates of ITP-CRISPR detection and DETECTR (2) detection of N gene, E gene, and RNase P genes on contrived samples. For each method and target gene, fluorescence is normalized to the maximum value across all samples. For the DETECTR method, we followed the protocol in Broughton et al. (2) and used endpoint fluorescence readout at 10 min.

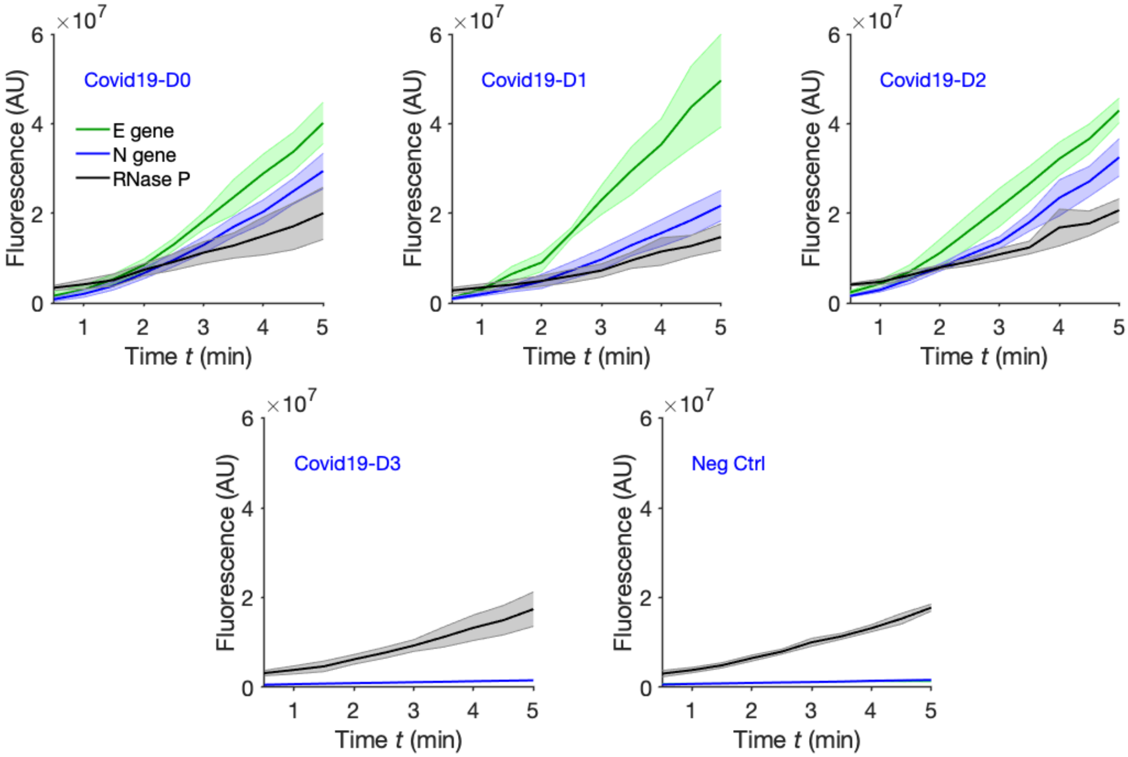

Figure S10. Measured kinetic curves for ITP-CRISPR detection of N gene, E gene, and RNase P gene on clinical samples. 1:10 serial dilutions (Covid19-D1 to D3) of a COVID-19 positive NP swab sample (Covid19-D0) and a pooled negative (Neg Ctrl) NP swab sample were tested in three technical replicates (n = 3). Serial dilutions for the positive samples were performed in VTM from pooled negative swab samples. Fluorescence signal was measured every 30 s. Solid lines represent the mean signal and the shaded envelope represents the standard deviation. Covid19-D3 was below the LOD of our assay as confirmed by RT-PCR (Fig. 2c).

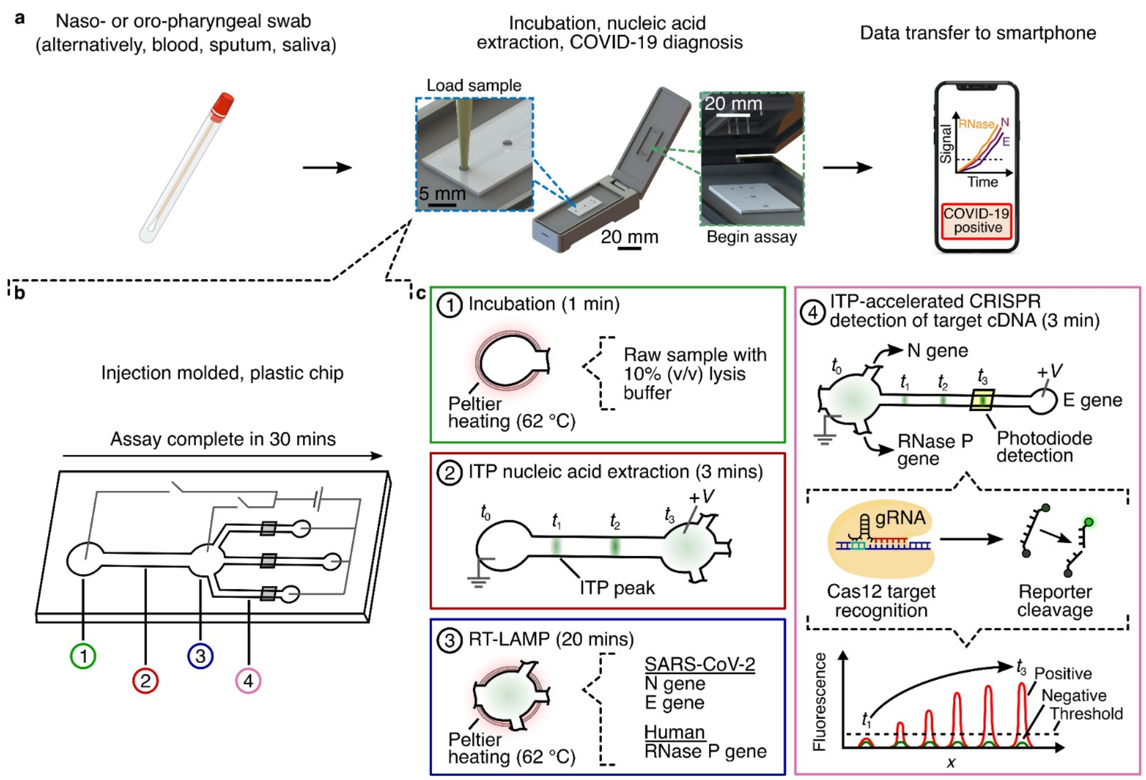

Figure S11. Schematic depiction of the concept of a fully integrated chip design on a portable device. An injection molded plastic chip could be loaded into a hand-held portable device powered and controlled via USB includes laser-induced fluorescence detector, DC-to-DC converters, a photodiode, and integrated microheaters (for LAMP incubation at 62°C). See Bercovici et al. (3) for an integration of at least one ITP assay into such a portable device. We hypothesize that such a system can integrate ITP-based nucleic acid extraction, multiplexed isothermal amplification of target cDNA of N and E genes of SARS-CoV-2 and RNase P control, followed by ITP-CRISPR-based cDNA detection in three separate channels using photodiodes.

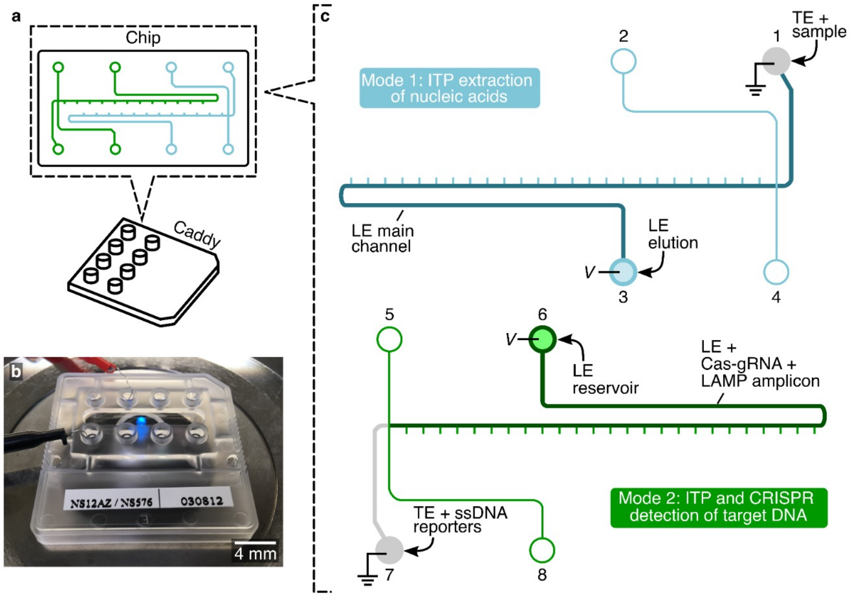

Figure S12. Experimental setup and microfluidic chip layout. (a) Schematic of the glass chip bonded to a plastic caddy using epoxy. The chip consists of two identical cross-channels adjacent to each other. (b) Experimental setup showing the chip and electrodes on a microscope stage during Mode 2 operation (c) Buffer placement and sample loading in Mode 1 and Mode 2. In Mode 1, first, reservoirs 2, 3, and 4 are filled with 10 µL of LE main channel buffer and vacuum is applied at reservoir 1 till the main channel is completely filled with LE. Then, reservoirs 3 and 1 are emptied and filled with 10 µL of LE elution buffer and lysed sample in TE, respectively. A constant voltage of 1 kV is applied for ~3 min. For Mode 2, first, 5 µL of LE combined with Cas12-gRNA and LAMP amplicon is loaded in reservoir 6, 5 µL of LE is loaded in reservoir 8, and 5 µL TE combined with ssDNA reporters is loaded in reservoir 7. Vacuum is applied at reservoir 5 briefly till the channels are filled as depicted in the schematic. Then, reservoirs 5, 6, and 8 are emptied and loaded with 10 µL of LE, and reservoir 7 is emptied and loaded with 10 µL TE. A constant current of 4 µA is applied for 5 min and fluorescence intensity of the ITP peak is recorded using a CMOS camera every 30 s.

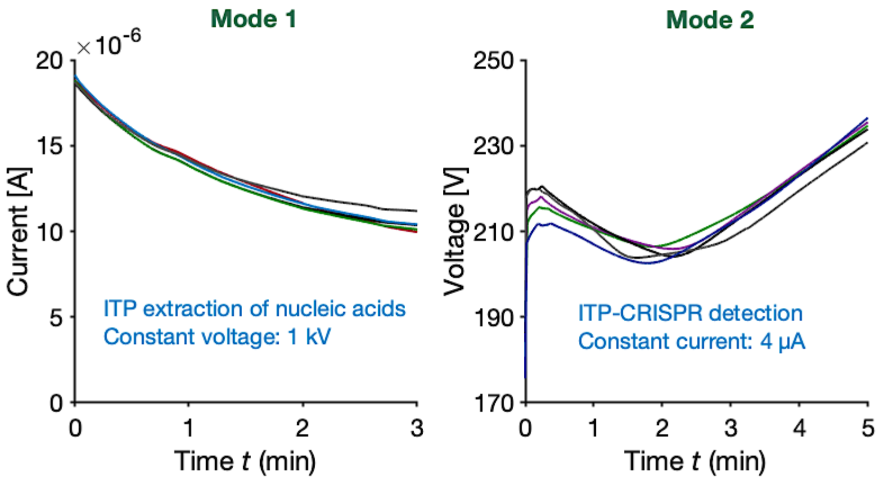

Figure S13. Measured current and voltage versus time during ITP extraction (Mode 1) and ITP-CRISPR detection (Mode 2). In operational Mode 1, a constant voltage of 1 kV was applied for 3 min to complete ITP extraction of nucleic acids from nasopharyngeal swab sample. In Mode 2, a constant current of 4 μA was applied for 5 min for ITP-CRISPR-based detection. Shown are current and voltage traces from five typical experiments to demonstrate the reproducibility of the process.

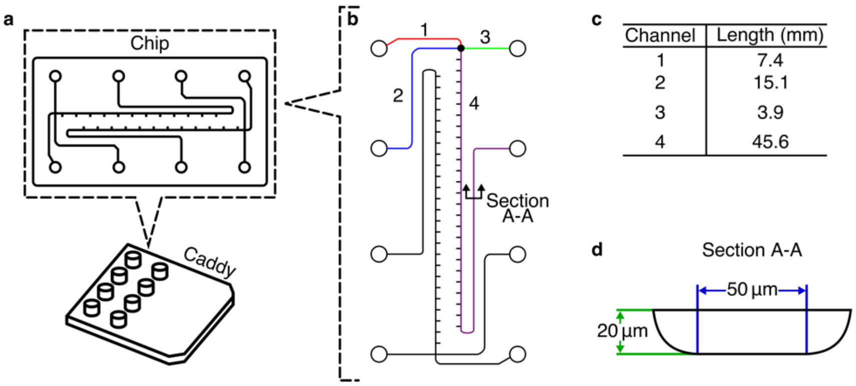

Figure S14. NS12AZ microfluidic chip geometry and fabrication. (a) Schematic of the microfluidic chip and caddy. The chip consists of two identical microfluidic channels which are adjacent and in opposite directions to each other. A plastic caddy consisting of eight reservoir ports is bonded to the glass chip using epoxy. NS12AZ chips were purchased from PerkinElmer, Inc. (parent company of Caliper Life Sciences). (b) Schematic of the top view of the channels. A solid black circle symbol represents the intersection of four channel segments (1 to 4). Ticks in channel section 4 are vertically separated by 1 mm. (c) Lengths of the four channel segments. (d) Cross-sectional view of the channel. The channels are wet-etched to a 20 µm depth using a 50 µm mask width, resulting in a channel width of 90 µm and a roughly D-shaped cross section.

Table S1. List of gRNAs, LAMP primers, RT-PCR primers, template and reporter sequences. Mtb sequences were used for ITP co-focusing experiments of Fig. 1c. E gene, N gene, and RNase P sequences (originally published in Broughton et al. (2)) were used for SARS-CoV-2 detection. The ssDNA reporter was used commonly for all ITP-CRISPR experiments.

LAMP primer	Sequence (5'-3')
N-gene F3	AAC ACA AGC TTT CGG CAG
N-gene B3	GAA ATT TGG ATC TTT GTC ATC C
N-gene FIP	TGC GGC CAA TGT TTG TAA TCA GCC AAG GAA ATT TTG GGG AC
N-gene BIP	CGC ATT GGC ATG GAA GTC ACT TTG ATG GCA CCT GTG TAG
N-gene LF	TTC CTT GTC TGA TTA GTT C
N-gene LB	ACC TTC GGG AAC GTG GTT
E-gene F3	CCG ACG ACG ACT ACT AGC
E-gene B3	AGA GTA AAC GTA AAA AGA AGG TT
E-gene FIP	ACC TGT CTC TTC CGA AAC GAA TTT GTA AGC ACA AGC TGA TG
E-gene BIP	CTA GCC ATC CTT ACT GCG CTA CTC ACG TTA ACA ATA TTG CA
E-gene LF	TCG ATT GTG TGC GTA CTG C
E-gene LB	TGA GTA CAT AAG TTC GTA C
RNaseP POP7 F3	TTG ATG AGC TGG AGC CA
RNaseP POP7 B3	CAC CCT CAA TGC AGA GTC
RNaseP POP7 FIP	GTG TGA CCC TGA AGA CTC GGT TTT AGC CAC TGA CTC GGA TC
RNaseP POP7 BIP	CCT CCG TGA TAT GGC TCT TCG TTT TTT TCT TAC ATG GCT CTG GTC
RNaseP POP7 LF	ATG TGG ATG GCT GAG TTG TT
RNaseP POP7 LB	CAT GCT GAG TAC TGG ACC TC
**qPCR primer**	**Sequence (5'-3')**
E_Sarbeco_F1	ACA GGT ACG TTA ATA GTT AAT AGC GT
E_Sarbeco_R2	ATA TTG CAG CAG TAC GCA CAC A
E_Sarbeco_P1	5 -FAM/ACA CTA GCC ATC CTT ACT GCG CTT CG/3 -BHQ-1
RP-F	AGA TTT GGA CCT GCG AGC G
RP-R	GAG CGG CTG TCT CCA CAA GT
RP-P	5 -FAM/TTC TGA CCT GAA GGC TCT GCG CG/3 -BHQ-1
**gRNA**	**Sequence (5'-3')**
E gene	UAA UUU CUA CUA AGU GUA GAU GUG GUA UUC UUG CUA GUU AC
N gene	UAA UUU CUA CUA AGU GUA GAU CCC CCA GCG CUU CAG CGU UC
RNase P	UAA UUU CUA CUA AGU GUA GAU AAU UAC UUG GGU GUG ACC CU
Mtb	UAA UUU CUA CUA AGU GUA GAU CCG CGG GUG GUC CCG GAC AG/3Cy5Sp/
**Template and reporter**	**Sequence (5'-3')**
ssDNA reporter	/56-FAM/TTATT/3IABkFQ/
Mtb-F template	ATC AGC GAT CGT GGT CCT GCG GGC TTT GCC GCG GGT GGT CCC GGA CAG GCC GAG TTT GGT CAT CAG CCG TTC
Mtb-R template	GAA CGG CTG ATG ACC AAA CTC GGC CTG TCC GGG ACC ACC CGC GGC AAA GCC CGC AGG ACC ACG ATC GCT GAT

Table S2. Test interpretation method based on possible outcomes of the ITP-CRISPR assay for SARS-CoV-2. Symbols '+' and '-' indicate positive and negative detection by the microfluidic ITP-CRISPR assay for the respective target genes (N, E, and RNase P). We interpret the sample to be SARS-CoV-2 positive if at least one of N gene or E gene is detected. A sample is interpreted as SARS-CoV-2 negative if none of N gene or E gene is detected and RNase P gene is detected. If none of N, E, or RNase P genes are detected, we interpret the test as "failed extraction", and this outcome will require extraction and testing processes to be repeated again before any conclusion can be drawn. Our test interpretation is similar to that Broughton et al. (2).

N gene	E gene	RNase P	Test interpretation
+	+	+/-	Positive
+	-	+/-	Positive
-	+	+/-	Positive
-	-	+	Negative
-	-	-	Failed extraction

Table S3. Ct values of the 64 patient samples corresponding to data presented in Fig. 3. Ct values are based on the WHO RT-PCR assay (4) implemented at the Stanford Clinical Virology Lab (5). The RT-PCR assay targets the viral E gene and uses RNase P as control. N.D: not detected.

Negative samples			Positive samples		
Sample	Ct (E gene)	Ct (RNase P)	Sample	Ct (E gene)	Ct (RNase P)
COVID19-N01	N.D.	23.91	COVID19-P01	30.97	24.84
COVID19-N02	N.D.	25.13	COVID19-P02	28.37	24.3
COVID19-N03	N.D.	21.69	COVID19-P03	24.1	23.23
COVID19-N04	N.D.	23.01	COVID19-P04	26.79	25.4
COVID19-N05	N.D.	24.29	COVID19-P05	24.01	20.69
COVID19-N06	N.D.	22.6	COVID19-P06	22.2	20.19
COVID19-N07	N.D.	22.15	COVID19-P07	16.7	19.63
COVID19-N08	N.D.	22.75	COVID19-P08	30.51	25.34
COVID19-N09	N.D.	20.95	COVID19-P09	32.22	23.51
COVID19-N10	N.D.	20.8	COVID19-P10	27.28	25.73
COVID19-N11	N.D.	21.59	COVID19-P11	15.62	19.88
COVID19-N12	N.D.	22.49	COVID19-P12	11.71	21.52
COVID19-N13	N.D.	20.63	COVID19-P13	32.56	23.02
COVID19-N14	N.D.	22.41	COVID19-P14	25.09	24.38
COVID19-N15	N.D.	21.07	COVID19-P15	28.23	23.73
COVID19-N16	N.D.	23.11	COVID19-P16	27.32	22.79
COVID19-N17	N.D.	22.25	COVID19-P17	23.58	23.39
COVID19-N18	N.D.	23.92	COVID19-P18	28.18	26.57
COVID19-N19	N.D.	22.57	COVID19-P19	30.89	26.86
COVID19-N20	N.D.	21.74	COVID19-P20	29	24.2
COVID19-N21	N.D.	22.17	COVID19-P21	25.96	24.67
COVID19-N22	N.D.	22.8	COVID19-P22	31.4	27.63
COVID19-N23	N.D.	24.32	COVID19-P23	30.95	26.14
COVID19-N24	N.D.	23.31	COVID19-P24	21.48	21.71
COVID19-N25	N.D.	21.19	COVID19-P25	28.95	26.53
COVID19-N26	N.D.	22.9	COVID19-P26	31.75	25.13
COVID19-N27	N.D.	25.21	COVID19-P27	30.78	23.79
COVID19-N28	N.D.	20.82	COVID19-P28	30.12	25.18
COVID19-N29	N.D.	22.17	COVID19-P29	24.39	23.77
COVID19-N30	N.D.	21.87	COVID19-P30	22.48	33.77
COVID19-N31	N.D.	19.76	COVID19-P32	35.75	22.38
COVID19-N32	N.D.	23.77	COVID19-P33	35.25	20.07

Movie S1. ITP visualization experiment shows the co-focusing of Cas12-gRNA (red) and ssDNA reporters (green) into a ~100 pL volume and preconcentration of CRISPR reagents by ~1,000-fold.

Movie S2. ITP-CRISPR detection of a SARS-CoV-2 positive control. Fluorescence of ITP peak significantly increased with time indicating cleavage of reporter ssDNA molecules by target-activated CRISPR-Cas12 enzyme. Time label has the format of minute:second.

Movie S3. ITP-CRISPR detection of a SARS-CoV-2 negative control. Minimal change in fluorescence signal of the ITP peak relative to the background signal. Time label has the format of minute:second.

SI References

1. M. Bercovici, S. K. Lele, J. G. Santiago, Open source simulation tool for electrophoretic stacking, focusing, and separation. *J. Chromatogr. A* (2009) https:/doi.org/10.1016/j.chroma.2008.12.022.
2. J. P. Broughton, *et al.*, CRISPR–Cas12-based detection of SARS-CoV-2. *Nat. Biotechnol.* (2020) https:/doi.org/10.1038/s41587-020-0513-4.
3. M. Bercovici, G. V. Kaigala, C. J. Baokhouse, J. G. Santiago, Fluorescent carrier ampholytes assay for portable, Label-Free detection of chemical toxins in tap water. *Anal. Chem.* (2010) https:/doi.org/10.1021/ac902526g.
4. V. M. Corman, *et al.*, Detection of 2019 novel coronavirus (2019-nCoV) by real-time RT-PCR. *Eurosurveillance* (2020) https:/doi.org/10.2807/1560-7917.ES.2020.25.3.2000045.
5. Accelerated Emergency Use Authorization (EUA) Summary SARS-CoV2 RT-PCR Assay (Stanford Health Care Clinical Virology Laboratory).
